# Surgical treatment of a giant tibial high-grade mixofibrosarcoma with preservation of limb function: a case report

**DOI:** 10.1186/1477-7800-6-16

**Published:** 2009-09-17

**Authors:** Domenico Marotta, Marina Angeloni, Marzia Salgarello, Maria Lucia Ricciardella, Byron Chalidis, Giulio Maccauro

**Affiliations:** 1 Department of Orthopaedic and Traumatology, Catholic University of Rome, Italy; 2 Department of Plastic Surgery, Catholic University of Rome, Italy; 3 Department of Orthopaedic and Traumatology, University of California, San Francisco, USA

## Abstract

Myxofibrosarcoma is one of the most common sarcomas in elderly patients showing a slight male prevalence. The tumor is mainly located in lower and upper extremities and rarely in trunk, neck and feet. We present a case of a 84-year-old man referred to our tumour center with a giant and neglected high-grade tibial myxofibrosarcoma in the anteromedial side of tibial mid-diaphysis. Large size lesions in association with older age may jeopardise the maintenance of limb vitality, vascularity and stability.

Authors performed a complete tumour resection, followed by reconstruction of bone and soft tissue defects with cement, plate and a musculocutaneous gastrocnemius flap, in order to cover the underlying bone and promote uneventful healing and perfusion of the operated extremity.

At 2 years postoperatively, limb salvage, good functional outcome and no tumour recurrence were reported while the patient was able to effectively perform the majority of the daily activities.

## Introduction

Myxofibrosarcoma is one of the most common sarcomas in elderly patients showing a slight male prevalence [1]. Tumor incidence is higher between the sixth and eighth decade of life and rarely the lesion can be seen in patients less than 20 year of age [2]. Myxofibrosarcoma was firstly described by Weiss and Enzinger as a myxoid variant of malignant fibrous histiocytoma (MFH) [1]. It comprises a spectrum of malignant fibroblastic lesions with variably myxoid stroma, pleomorphism and distinctively curvilinear vascular pattern [3,4]. The tumor is mainly located in lower (48%) and upper extremities (34%) and rarely in trunk, neck and feet.

Wide resection of the malignant tumour constitutes the preferred treatment choice [5]. However, large size lesions in association with older age may jeopardise the maintenance of limb vitality, vascularity and stability. Therefore, musculocutaneous flaps are frequently applied to cover the underlying bone and promote uneventful healing and perfusion of the operated extremity [6]. We present a case with a giant and neglected high-grade tibial myxofibrosarcoma. Complete tumour resection was followed by reconstruction of bone and soft tissue defects with cement and a musculocutaneous gastrocnemius flap, respectively. At 2 years postoperatively, limb salvage, good functional outcome and no tumour recurrence were reported while the patient was able to effectively perform the majority of the daily activities.

## Case presentation

**Figure 1:**
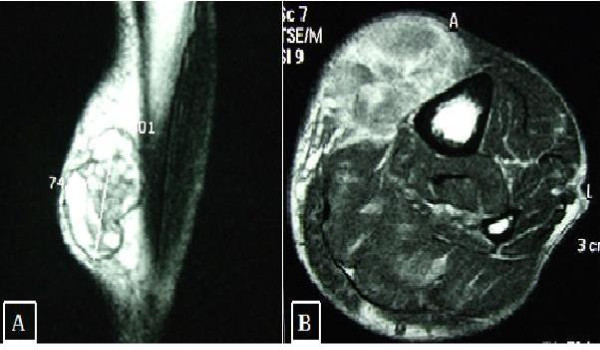
**MRI images of the lesion**. A) Sagittal TSE sequence with deletion of the T2 signal from adipose tissue. B) Axial MRI performed with T2 gradient-eco sequence.

**Figure 2:**
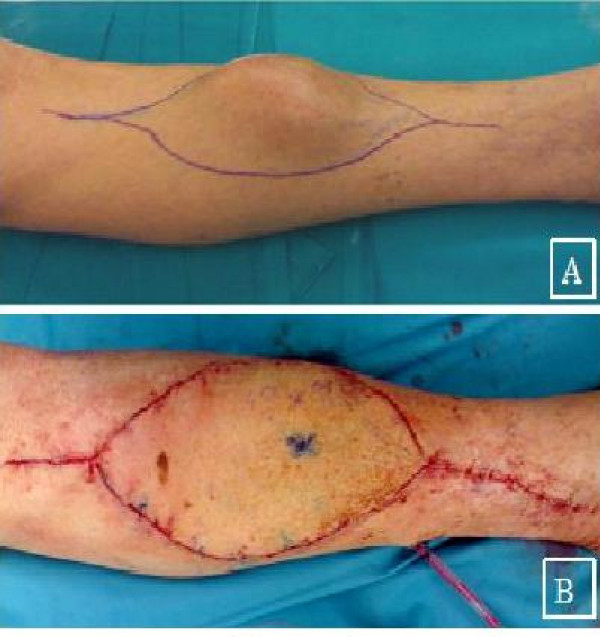
**Clinical assessment**. A) Clinical presentation. B) Postoperative evaluation: coverage of soft tissue and skin defect with a micro vascular musculocutaneous flap.

An 84-year-old man was referred to our tumour center with a large mass (8 × 5 cm) in the anteromedial side of tibial mid-diaphysis (Fig. 1). The lesion was painless and the patient reported a rapid increase in its size during the last year. Before that time, he did not worry of its nature, as it was remained unchanged for many years. Plane X-Rays showed a radiolucent lesion adjacent to tibial bone without cortical disruption. Magnetic Resonance Imaging (MRI) revealed an ovular plurilobulated and inhomogeneous soft-tissue mass with a predominant semisolid matrix (Fig. 1). Some necrotic as well as adipose tissue areas were also recognised. However, the tumour did not infiltrate the bone or the surrounding muscles (Fig. 2). The patient had hypertension and diabetes but his general health was overall good.

The next step was fine needle aspiration biopsy of the mass. Histological examination revealed that the tumour was a malignant myxofibrosarcoma. Chest and abdominal Computed Tomography (CT) scans as well as radionuclide total bone scan excluded the presence of systemic lesions or metastasis. Therefore, the patient was scheduled to undergo surgical resection of the lesion with additional removal of the adjacent soft tissue structures.

Under general anaesthesia and after tourniquet inflation, *en block *wide excision of the mass was performed. As the tumour was in close proximity to mid-diaphyseal tibial bone, part of the tibial anterior-medial cortex was also removed to facilitate at least 2 cm tumour-free resection margins circumferentially. The tibial defect of 9 cm was filled with acrylic cement and fixed with a 4.5 mm locking plate and screws. Tumour removal was followed by coverage of the large remaining soft tissue and skin defect (25 cm) with a microvascular musculocutaneous flap from the contralateral thigh.

Histological analysis of the excised tumour showed a high-grade myxofibrosarcoma with solid sheets and areas of necrosis. At higher magnification, multiple spindle cell cells of mesenchymal origin surrounded by many polygonal epithelioid cells with pleomorphism and elevated mitotic index were found. Surgical margins were disease free. In addition, immunohistochemical staining was negative for CD34, smooth muscular actin, for desmin, keratins, caldesmon, and S-100, which confirmed the high-grade character of the lesion.

**Figure 3:**
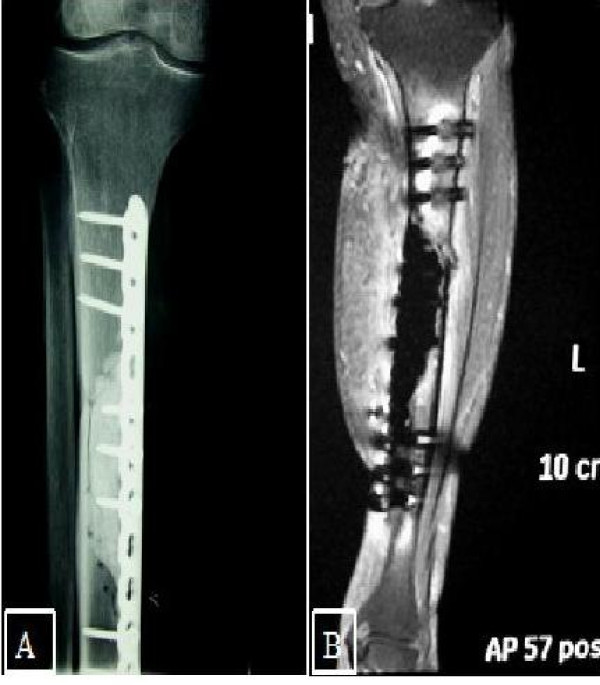
**Radiological findings at 2 years follow up**. A) Anteroposterior XRay. B) Sagittal T2 MRI.

The postoperative period was uneventful. Partial weight bearing for 6 weeks was advised. After wound healing, about 3 weeks, the patient was referred to the Oncology Department for local Radioterapy. At 2 years follow up evaluation, plain X-Rays and MRI of the operated site didn't reveal any signs of local recurrence (Fig. 3). Furthermore, chest and abdominal CT scans were negative for metastasis. The flap looked intact and well vascularised. The patient could stand and walk without any restriction, he did not report any residual pain and he was able to perform his usual daily activities.

## Discussion

Myxofibrosarcoma includes a group of mesenchymal neoplasms of fibrous-histiocytic malignant line that mainly is appeared in extremities and has a high rate of local recurrence. The tumour shows a wide variability of presentation and prognosis and is classified as low or high grade [1]. Low-grade lesions are characterized by mixoid matrix as well as low percentage of stellate and spindled cells with elongated capillaries. Conversely, multinucleated giant cells, solid sheets and cellular fascicle of tumour cells with haemorrhagic and necrotic areas are usually apparent in high-grade myxobibrosarcomas [2].

About 2/3 of lesions are superficial and developed in dermal or subcutaneous tissue [1]. Superficially or subcutaneously located tumours consist of multiple gelatinous or firmer nodules that often spread in a longitudinal manner. Furthermore, they usually have infiltrative margins and extended beyond what is detected clinically. On the other hand, deep-seated intramuscular lesions are mainly subfascial and they show a more infiltrative growth pattern. Moreover, they are predominantly high-grade malignant tumours as the relevant incidence may be raised up to 76% of cases [2].

Local recurrence occurs in approximately 50-60% of cases regardless the histological grade and location of the lesion [1,2]. However, suboptimal resection without tumour-free surgical margins may increase the recurrence rate of the myxofibrosarcoma [7]. Although distant osseous, lung and lymph node metastases are rare in low-grade tumours, they may be apparent in about 20-35% of high-grade myxofibrosarcomas. Local recurrence and metastases seem to be non-independent factors as frequent local recurrence could increase the risk of distant expansion of the tumour. Overall, the 5-year survival rate is around 60-70% [2]

Wide resection of the mass is mandatory to decrease the risk of local recurrence and increase the overall survival rate. [3] This may cause a significant bone, muscular or skin defect particularly in superficially locate myxofibrosarcomas. Masono et al illustrated the high rate of local failure when an unplanned excision of a soft tissue sleeve was taken place. In the presented case, *en block *wide excision of the mass with tumor-free margins was achieved by partial removal of the anteromedial tibial cortex. Bone reconstruction was performed with plate, screws and acrylic cement allowing early mobilisation and weight-bearing. In our clinic we usually use this solution for the reconstruction of bone defects, because of stability and strength regained to the bone.

However, the large residual soft tissue defect in association with patient's age threatened the integrity and function of the limb. Coverage of the large soft tissue defect was performed with a musculo-cutaneous vascular flap that was harvested from the contralateral thigh [6]. Although the procedure is indicated in patients without serious co morbidities, which may affect limb perfusion, it may lead to good result even in extreme conditions. The latter were presented in our case and included advanced age of the patient (> 80 years), hypertension and diabetes. As the alternative solution in similar circumstances is only limb amputation, all efforts should be made to preserve the limb and restore its function.

Postoperative adjuvant radiation therapy is usually applied even if surgical margins are tumour-free in order to optimise the benefits obtained by the surgery, as universally accepted in literature[1,3,5,8]. Manoso et al however, declares that radiation therapy cannot replace surgical treatment or reduce local recurrence. Manoso's results showed no appreciable effect on local recurrence, referring local recurrence to inadequate surgery. The local failure rate in patients with microscopically positive margins, who received radiotherapy without surgery was 100%, as compared with 50% failure rate for patients who underwent a second re-excision to negative margins and radiotherapy.

## Conclusion

Giant myxofibrosarcomas may severely compromise limb integrity and function. Wide resection and reconstruction of the remaining soft-tissue defect with a micro-vascular flap can be an effective limb-sparing procedure even in elderly with concomitant medical co morbidities.

## Consent

Written consent was obtained from the patient for publication of this case report.

## Competing interests

The authors declare that they have no competing interests.

## Authors' contributions

DM prepared the draft of case report. GM conceived the idea of the case report and helped with the draft of it. BC, MA, MS and MLR helped the draft of the case report. All authors read and approved the final manuscript.
